# *VPS35* D620N knockin mice recapitulate cardinal features of Parkinson’s disease

**DOI:** 10.1111/acel.13347

**Published:** 2021-03-21

**Authors:** Mengyue Niu, Fanpeng Zhao, Karina Bondelid, Sandra L. Siedlak, Sandy Torres, Hisashi Fujioka, Wenzhang Wang, Jun Liu, Xiongwei Zhu

**Affiliations:** ^1^ Department of Neurology and Institute of Neurology Ruijin Hospital Affiliated to Shanghai Jiao Tong University School of Medicine Shanghai China; ^2^ Department of Pathology Case Western Reserve University Cleveland OH USA; ^3^ Electron Microscopy Core Facility Case Western Reserve University Cleveland OH USA

**Keywords:** mitochondria dynamics, neurodegeneration, Parkinson's disease, VPS35, α‐synuclein

## Abstract

D620N mutation in the *vacuolar* *protein* *sorting* *35* *ortholog* (VPS35) gene causes late‐onset, autosomal dominant familial Parkinson's disease (PD) and contributes to idiopathic PD. However, how D620N mutation leads to PD‐related deficits in vivo remains unclear. In the present study, we thoroughly characterized the biochemical, pathological, and behavioral changes of a *VPS35* D620N knockin (KI) mouse model with chronic aging. We reported that this *VPS35* D620N KI model recapitulated a spectrum of cardinal features of PD at 14 months of age which included age‐dependent progressive motor deficits, significant changes in the levels of dopamine (DA) and DA metabolites in the striatum, and robust neurodegeneration of the DA neurons in the SNpc and DA terminals in the striatum, accompanied by increased neuroinflammation, and accumulation and aggregation of α‐synuclein in DA neurons. Mechanistically, D620N mutation induced mitochondrial fragmentation and dysfunction in aged mice likely through enhanced VPS35‐DLP1 interaction and increased turnover of mitochondrial DLP1 complexes in vivo. Finally, the *VPS35* D620N KI mice displayed greater susceptibility to MPTP‐mediated degeneration of nigrostriatal pathway, indicating that *VPS35* D620N mutation increased vulnerability of DA neurons to environmental toxins. Overall, this *VPS35* D620N KI mouse model provides a powerful tool for future disease modeling and pharmacological studies of PD. Our data support the involvement of VPS35 in the development of α‐synuclein pathology in vivo and revealed the important role of mitochondrial fragmentation/dysfunction in the pathogenesis of *VPS35* D620N mutation‐associated PD in vivo.

## Graphical abstract

Homozygous *VPS35* D620N KI mice recapitulate cardinal features of PD including motor deficits, dopaminergic neuronal loss, and reduced dopamine along with accumulation and aggregation of α‐synuclein in an age‐dependent manner. *VPS35* D620N mutation induced mitochondrial fragmentation and dysfunction through enhanced VPS35‐DLP1 interaction and increased turnover of mitochondrial DLP1 complexes in vivo. *VPS35* D620N KI mice displayed greater susceptibility to PD‐related neurotoxin of MPTP.

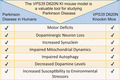

## 1 INTRODUCTION

Parkinson's disease (PD) is the most common neurodegenerative movement disorder characterized by the degeneration of dopaminergic (DA) neurons in the substantia nigra pars compacta (SNpc) and Lewy body pathology development (Lang & Lozano, 1998). The pathogenesis of PD is considered to be multifactorial, including genetic and environmental factors together with age (De Lau & Breteler, 2006). Although PD typically occurs in a sporadic manner, approximately 5% to 10% of patients have a positive family history of PD and dozens of genes have been found to be affected in those PD stricken families (Lesage & Brice, 2009). For instance, the mutational hot spot D620N substitution in *vacuolar* *protein* *sorting* *35* (VPS35) was reported to cause late‐onset autosomal dominant PD (Vilariño‐Güell et al., 2011; Zimprich et al., 2011) and has been identified in multiple familial PD cases, as well as sporadic patients, across different ethnic populations (Williams et al., 2017). The frequency of the D620N mutation is estimated to be about 1.5% in familial PD, representing the second most common cause of late‐onset autosomal dominant PD after *LRRK2* (Deng et al., 2013). Patients with *VPS35* D620N mutation resemble typical idiopathic disease with bradykinesia, resting tremor, and good response to levodopa therapy (Zimprich et al., 2011).

Extensive efforts have been devoted to understanding the pathogenic mechanisms of VPS35 mutations in PD, especially since it is highly expressed in DA neurons and plays a critical role in the survival and maintenance of DA neurons (Tang, Liu, et al., 2015). VPS35 is a key component of the heteropentameric mammalian retromer complex which mediates retrograde transport of cargo proteins from endosome to Golgi, endosome to plasma membrane, and mitochondria to the peroxisomes or lysosome (Bonifacino & Hurley, 2008; Braschi et al., 2010; Hierro et al., 2007; Seaman, 2004, 2012). Retromer consists of a VPS35‐VPS29‐VPS26 cargo recognition subcomplex and a SNX‐BAR dimer. Although the *VPS35* D620N mutation does not change the stability and assembly of VPS35‐VPS26‐VPS29 subcomplex, it alters cargo recognition of certain substrates and causes alterations in retromer‐mediated trafficking of several proteins along different pathways in either a gain‐of‐function or loss‐of‐function manner. On one hand, *VPS35* D620N mutation causes reduced interaction between retromer and the Wiskott–Aldrich syndrome and SCAR homolog (WASH) complex which thus selectively disrupts endosome‐to‐plasma membrane sorting of select cargos such as ATG9A, and causes impaired autophagy in a partial loss‐of‐function manner in Hela cells and SH‐SY5Y cells (Zavodszky et al., 2014). *VPS35* D620N mutation can also impair endosome‐to‐TGN retrieval of select cargos such as the cation‐independent mannose‐6‐phosphate receptor (CI‐M6PR) and LAMP2a and cause lysosomal or autophagy deficits in vitro (Follett et al., 2014; Tang, Erion, et al., 2015). On the other hand, *VPS35* D620N mutation can increase VPS35 interaction with mitochondrial DLP1 complexes and cause excessive mitochondrial fission and mitochondrial dysfunction through enhanced recycling of mitochondrial DLP1 complex in neuronal cells and fibroblasts from PD patients bearing *VPS35* D620N mutation (Wang et al., 2016). These in vitro studies suggest that VPS35 mutation likely produces subtle functional deficits depending on the specific phenotypic or cellular context. Therefore, it is of great importance to investigate the pathogenic mechanism of *VPS35* D620N mutation in vivo.

To elucidate the pathophysiological changes of *VPS35* D620N mutation with chronic aging, we utilized the *VPS35* D620N knockin (KI) mouse model to facilitate the study of *VPS35* D620N mutant at its physiological expression pattern and level in vivo, which represents a more accurate disease model. After thorough characterization, we demonstrated that *VPS35* D620N KI mice recapitulated many of the cardinal features of PD in an age‐dependent manner and provided evidence that D620N mutant caused mitochondrial fragmentation and dysfunction in vivo. We further demonstrated that *VPS35* D620N KI mice have increased susceptibility to the PD‐related toxic insult MPTP providing evidence of the interaction between genetic factors and environmental toxins in the development of PD.

## 2 RESULTS

### 2.1 Progressive motor deficits in *VPS35* D620N KI mice

The constitutive *VPS35* D620N KI mice were obtained from The Jackson Labs (Stock No: 023409) and were originally developed by crossing conditional *VPS35* D620N KI mice (Stock No: 021807) with Sox2‐Cre‐delete line (Stock No: 008454) by the Michael J. Fox Foundation as recently reported (Cataldi et al., 2018). Intercrosses of constitutive heterozygous *VPS35* D620N KI mice were performed to produce *VPS35^WT^* ^/^ *^WT^*, *VPS35^D620N^* ^/^ *^WT^*, and *VPS35^D620N^* ^/^ *^D620N^* littermate mice. D620N mutation of KI mice were verified by genomic sequencing (Figure S1a) and PCR genotyping (Figure S1b). Consistent with previous report (Cataldi et al., 2018), both heterozygous (*VPS35^D620N^* ^/^ *^WT^*) and homozygous *VPS35* D620N KI mice (*VPS35^D620N^* ^/^ *^D620N^*) were viable and fertile, bred well to produce different genotype pups at expected frequency. Expression patterns of VPS35, VPS26, and VPS29 were not affected by the mutation in either VM (ventral midbrain (VM) or Striatum (STR) extracts (Figure S1c). There were no body weight differences between different genotypes in young mice as reported before (Cataldi et al., 2018), but a decreased body weight in male *VPS35^D620N^* ^/^ *^D620N^* mice compared with male WT control mice was noted starting at 10 months of age which became significant after 13 months of age (Figure S1d). No difference in the body weight was found in female mice though (Figure S1d).

**FIGURE 1:**
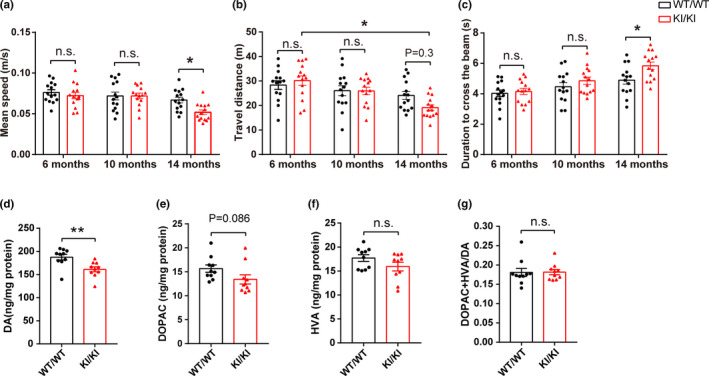
Behavioral and neurochemical characterization of *VPS35* D620N KI mice. (a) Mean speed in the open field test. (b) Total travel distance in open field test. (c) Duration to cross the narrow beam (9 mm) in beam walking test. Dots in figure histograms indicate single animals (the same with other figures). (*n* = 13–14/group, two‐way ANOVA with Tukey's post hoc test). (d–g) Concentrations of DA (d), DOPAC (e), HVA (f) and DA turnover rate (DOPAC+HVA/DA) (g) in *VPS35^D620N^* ^/^ *^D620N^* mice (KI/KI) compared with WT littermate control mice (WT/WT) at age of 15–16 months (*n* = 9–10/group, Student's *t* test, unpaired, two‐tailed). Data are shown as mean ± *SEM*; *p < 0.05; **p < 0.01; n.s., not significant

To evaluate the impact of *VPS35* D620N mutation on motor function, *VPS35^D620N^* ^/^ *^D620N^* mice (also referred to as *VPS35* D620N KI mice thereafter) and their WT littermate controls were subjected to various motor function tests including open field, beam walking, rotarod, and grip strength at three different ages (i.e., 6, 9–10, and 14 months). In open field test to evaluate general locomotor activity, no difference was found in younger groups (6 or 10 months) (Figure 1a,b). However, 14‐month *VPS35^D620N^* ^/^ *^D620N^* mice showed a significant reduction in the mean speed compared with age‐matched WT controls (two‐way ANOVA, *p* = 0.042) (Figure 1a). They also demonstrated decreased total travel distance compared with age‐matched WT controls although this did not reach significance (Figure 1b). In fact, *VPS35^D620N^* ^/^ *^D620N^* mice demonstrated progressive motor deficits since 14‐month group showed significantly decreased travel distance compared with 6‐month group (two‐way ANOVA, *p* = 0.011) while WT controls did not show similarly progressive deterioration (Figure 1b). In the beam walking test to assess fine motor coordination and balance, 14‐month *VPS35^D620N^* ^/^ *^D620N^* mice needed significantly longer time to cross the 9 mm beam compared with age‐matched WT controls (two‐way ANOVA, *p* = 0.041) (Figure 1c). No differences between *VPS35^D620N^* ^/^ *^D620N^* and WT controls were found in the rotarod or grip strength test (Figure S2a‐c).

Clinically, some non‐motor symptoms associated with PD may precede the onset of motor symptoms (Chaudhuri et al., 2006), which include hyposmia, gastrointestinal dysfunction, and sleep abnormalities. Buried pellet test and 1‐h stool collection were carried out to measure olfactory and gastrointestinal function, respectively, but no difference was noted between *VPS35^D620N^* ^/^ *^D620N^* and WT controls at any ages tested (Figure S2d‐f).

### 2.2 Decreased levels of dopamine in the STR of *VPS35* D620N KI mice

Parkinson's disease motor symptoms are due to the depletion of DA in the STR. Levels of DA and its metabolites DOPAC and HVA were measured in the STR of 15‐ to 16‐month‐old mice by HPLC analysis (Figure 1d–g). Consistent with impaired motor function, levels of DA were significantly decreased in *VPS35^D620N^* ^/^ *^D620N^* mice compared with WT controls (unpaired *t* test, *p* = 0.005) (Figure 1d). There were trends toward decreased levels of both DOPAC and HVA in *VPS35^D620N^* ^/^ *^D620N^* mice but they did not reach significance (Figure 1e,f). No difference in DA turnover rate (DOPAC+HVA/DA) was found between *VPS35^D620N^* ^/^ *^D620N^* and WT mice (Figure 1g).

### 2.3 Dopaminergic neurodegeneration in the nigrostriatal pathway of *VPS35* D620N KI mice

**FIGURE 2:**
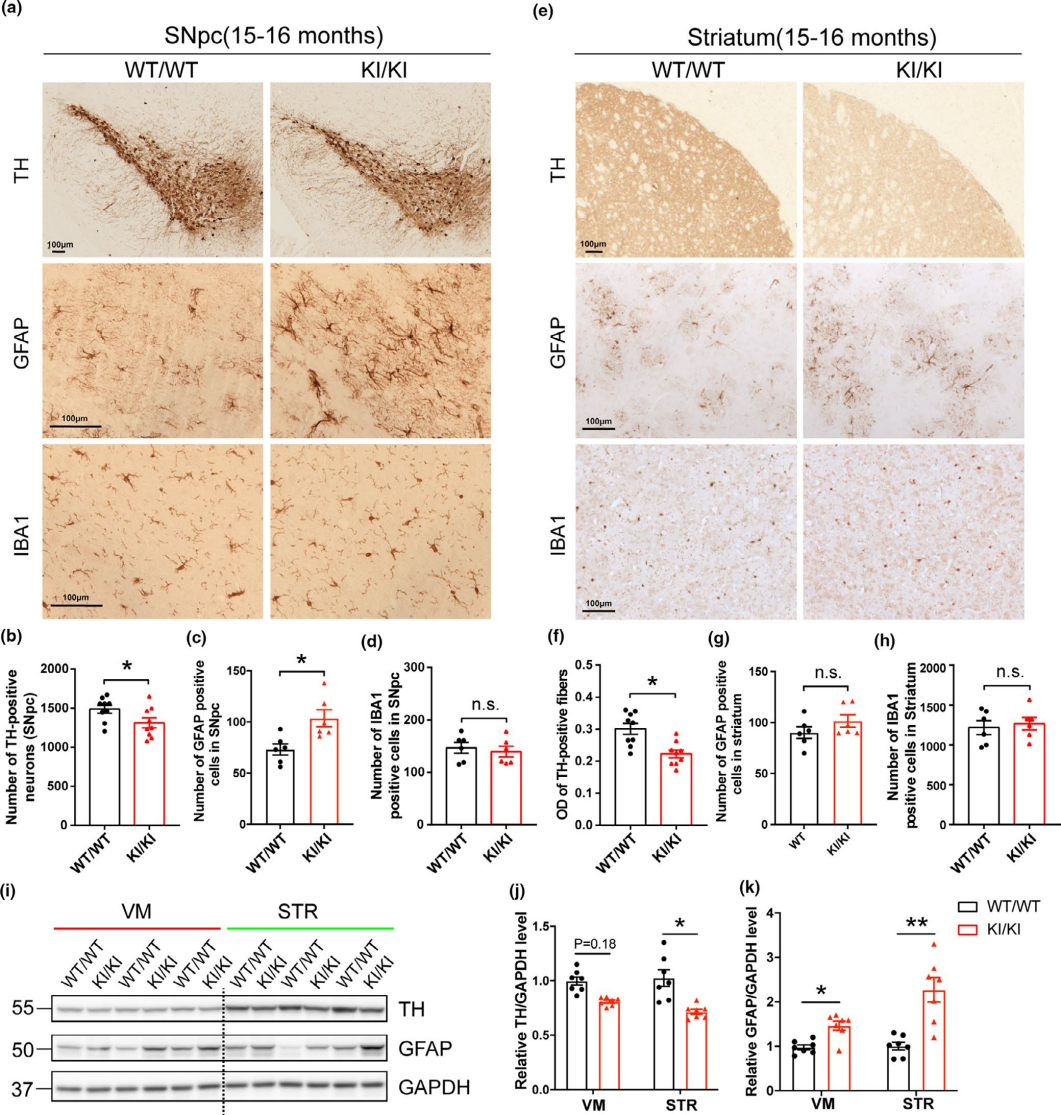
Neuropathological changes in *VPS35* D620N KI mice. (a,e) Representative images of TH, GFAP and IBA1 immunostaining in SNpc (a) and STR (e) of 15‐ to 16‐month‐old *VPS35^D620N^* ^/^ *^D620N^* and WT mice (*n* = 6–9/group). Scale bar, 100 μm. Quantification of TH‐positive neurons (b), GFAP‐positive cells (c) and IBA1‐positive cells (d) in SNpc. Quantification of optical density (OD) of TH‐positive fibers (f), GFAP‐positive cells (g), and IBA1‐positive cells (h) in STR. (i–k) Representative Western blots (i) and quantification of TH (j) and GFAP (k) in VM and STR extracts from 15‐ to 16‐month‐old *VPS35^D620N^* ^/^ *^D620N^* mice and WT controls (*n* = 7/group). Student's *t* test, unpaired, two‐tailed; data are shown as mean ± *SEM*; *p < 0.05; **p < 0.01; n.s., not significant

To determine whether the expression of D620N mutant induced degeneration of the nigrostriatal DA pathway in vivo, the substantia nigra (Figure 2a–d) and STR (Figure 2e–h) of *VPS35^D620N^* ^/^ *^D620N^* mice and WT control mice were examined at three different ages (6, 10, and 15–16 months). No changes in either the number of TH‐positive DA neurons in SNpc or the OD of TH‐positive fibers in STR were found in younger *VPS35^D620N^* ^/^ *^D620N^* mice (6 or 10 months) compared with their age‐matched WT controls (Figure S3a‐e). However, a significant loss of TH‐positive DA neurons (~12%) was detected in the SNpc of 15‐ to 16‐month‐old *VPS35^D620N^* ^/^ *^D620N^* mice compared with age‐matched WT controls (unpaired *t* test, *p* = 0.042) (Figure 2a,b). A significant reduction (~25%) in TH‐positive DA nerve terminals in STR was also found in 15‐ to 16‐month‐old *VPS35^D620N^* ^/^ *^D620N^* mice (unpaired *t* test, *p* = 0.011) (Figure 2e,f). Consistently, Western blot analysis revealed significantly reduced expression of TH (unpaired *t* test, *p* = 0.013) in the STR and a trend toward reduction in the VM in 15‐ to 16‐month‐old *VPS35^D620N^* ^/^ *^D620N^* mice (Figure 2i,j). The heterozygous (*VPS35^D620N^* ^/^ *^WT^*) mice did not demonstrate any significant changes in either TH‐positive DA neurons in the SNpc or OD of TH‐positive DA fibers in the STR compared with WT controls at all ages tested up to 16 months (Figure S4a–c).

Progressive neuroinflammation accompanies neurodegeneration in PD. At the age of 15–16 months, *VPS35^D620N^* ^/^ *^D620N^* mice exhibited significantly increased astrogliosis as evidenced by GFAP immunostaining in SNpc (unpaired *t* test, *p* = 0.012) (Figure 2a,c) but not in STR compared with WT controls (Figure 2e,g). Western blot analysis confirmed significantly increased GFAP expression in both VM (unpaired *t* test, *p* = 0.014) and STR (unpaired *t* test, *p* = 0.004) (Figure 2i,k). However, no microgliosis as measured by IBA1 immunostaining was found in SNpc (Figure 2a,d) or STR (Figure 2e,h) in *VPS35^D620N^* ^/^ *^D620N^* mice. In 6‐ and 10‐month‐old *VPS35^D620N^* ^/^ *^D620N^* and WT mice, neither astrogliosis nor microgliosis was found in SNpc or STR (Figure S3f‐n).

### 2.4 Accumulation and aggregation of α‐synuclein in *VPS35* D620N KI mice

**FIGURE 3:**
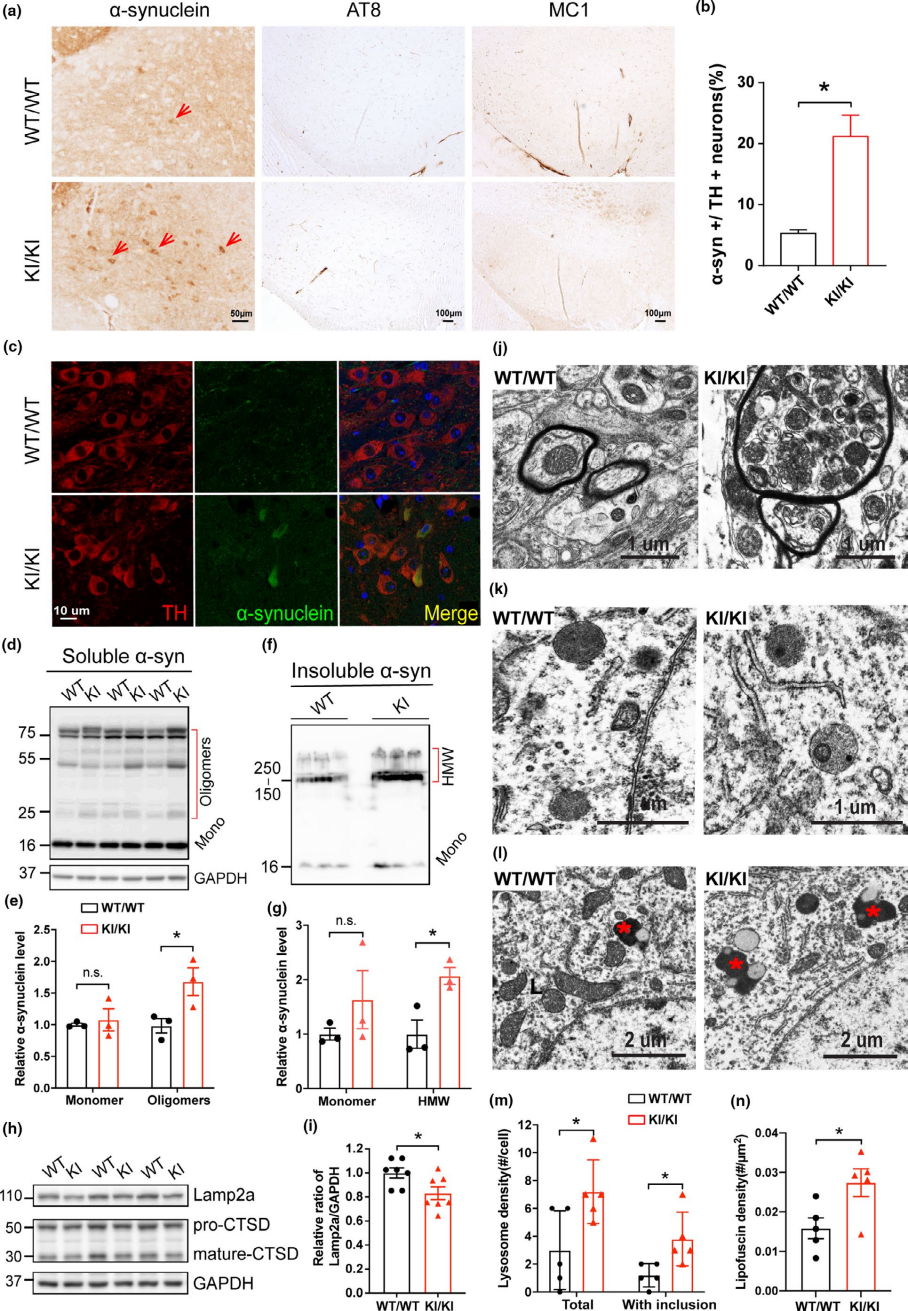
Accumulation and aggregation of α‐synuclein in *VPS35* D620N KI mice. (a) Representative images show immunohistochemical staining of α‐syn in SNpc from 15‐ to 16‐month‐old *VPS35^D620N^* ^/^ *^D620N^* mice and WT controls. Scale bar, 50 μm. (b) Quantification of α‐syn somatic accumulation in total TH^+^ neurons (%) in SNpc (*n* = 4; unpaired *t* test). (c) Confocal microscopy demonstrated colocalization of α‐syn (Green) and TH (Red) in SNpc from 15‐ to 16‐month‐old *VPS35^D620N^* ^/^ *^D620N^* mice and WT controls. (d–e) Western blots (d) and quantification (e) of α‐syn monomers and oligomers in Triton‐soluble fractions in VM from 15‐ to 16‐month‐old *VPS35^D620N^* ^/^ *^D620N^* mice and WT controls (*n* = 3; two‐way ANOVA with Tukey's post hoc test). (f–g) Western blots (f) and quantification (g) of α‐syn monomers and HMW bands in Triton‐insoluble fractions in VM from *VPS35^D620N^* ^/^ *^D620N^* mice and WT controls (*n* = 3; unpaired *t* test). (h–i) Representative Western blots (h) and quantification (i) of Lamp2a and Cathepsin D in VM from *VPS35^D620N^* ^/^ *^D620N^* mice and WT controls (*n* = 7; unpaired *t* test). (j) Representative electron microscopic image of dystrophic myelinated axons with autophagic vesicles in SNpc from 14‐month‐old *VPS35^D620N^* ^/^ *^D620N^* mice. (k) Lysosomes in SNpc neurons from 14‐month‐old WT with few dense bodies and from *VPS35^D620N^* ^/^ *^D620N^* mice with frequent inclusions, and quantification (m) (*n* = 5, unpaired *t* test). (l) Lipofuscins in SNpc neurons from 14‐month‐old WT and *VPS35^D620N^* ^/^ *^D620N^* mice, and quantification (n) (*n* = 5, unpaired *t* test, asterisk indicates lipofuscin). Data are shown as mean ± *SEM*; *p < 0.05, n.s., not significant

To further evaluate the impact of mutant VPS35 on PD‐related pathology (Spillantini et al., 1997), the expression of α‐syn was determined by immunohistochemistry and dramatically increased immunoreactivity of somatic α‐syn was found in SNpc of 15‐ to 16‐month‐old *VPS35^D620N^* ^/^ *^D620N^* mice compared with age‐matched WT controls (unpaired *t* test, *p* = 0.01) (Figure 3a,b). However, we did not find any tau pathology stained in the SNpc area by either AT8 or MC‐1 (Figure 3a), nor in the cortex (not shown). Confocal analysis of immunofluorescent studies confirmed that increased α‐syn almost completely localized to TH‐positive DA neurons (Figure 3c). To examine whether increased accumulation of intraneuronal α‐syn correlates with the formation of α‐syn oligomers and aggregates in vivo, we measured the levels of α‐syn protein in detergent‐soluble (1% Triton X‐100) and detergent‐insoluble extracts from the VM of 15‐ to 16‐month‐old *VPS35^D620N^* ^/^ *^D620N^* mice. Western blotting showed a significant increase in α‐syn oligomers in the Triton‐soluble fractions of VM from *VPS35^D620N^* ^/^ *^D620N^* mice compared with WT controls (unpaired *t* test, *p* = 0.036), whereas no difference in the levels of α‐syn monomers was found between these two groups (Figure 3d,e). Furthermore, levels of α‐syn aggregates as assessed by intensity of high‐molecular‐weight (HMW) bands were also increased in Triton‐insoluble fractions from *VPS35^D620N^* ^/^ *^D620N^* mice (unpaired *t* test, *p* = 0.024) (Figure 3f,g).

Previous studies suggest that *VPS35* D620N mutation may disrupt the retrieval of Lamp2a and cathepsin D, both of which are critical for α‐syn degradation (Follett et al., 2014; Tang, Erion, et al., 2015). Here, we found the levels of Lamp2a, but not Cathepsin D, were significantly decreased in VM from *VPS35^D620N^* ^/^ *^D620N^* mice (unpaired *t* test, *p* = 0.029) (Figure 3h,i), which suggests an impaired autophagy. Electron microscopic analysis found frequent dystrophic myelinated axons containing autophagic vacuoles in the SNpc from 14‐month‐old *VPS35^D620N^* ^/^ *^D620N^* mice, and these structures were not present in the WT mice (Figure 3j). Neurons in the SNpc from 14‐month‐old *VPS35^D620N^* ^/^ *^D620N^* mice had significantly more lysosomes than neurons in the WT mice (unpaired *t* test, *p* = 0.032), and these lysosomes often contained dense bodies or other inclusions rarely seen in the WT mice (unpaired *t* test, *p* = 0.023) (Figure 3k,m). In addition, lipofuscins were significantly increased in SNpc from 14‐month‐old KI mice compared with WT controls (unpaired *t* test, *p* = 0.03) (Figure 3l,n).

### 2.5 Mitochondrial deficits in *VPS35* D620N KI mice

**FIGURE 4:**
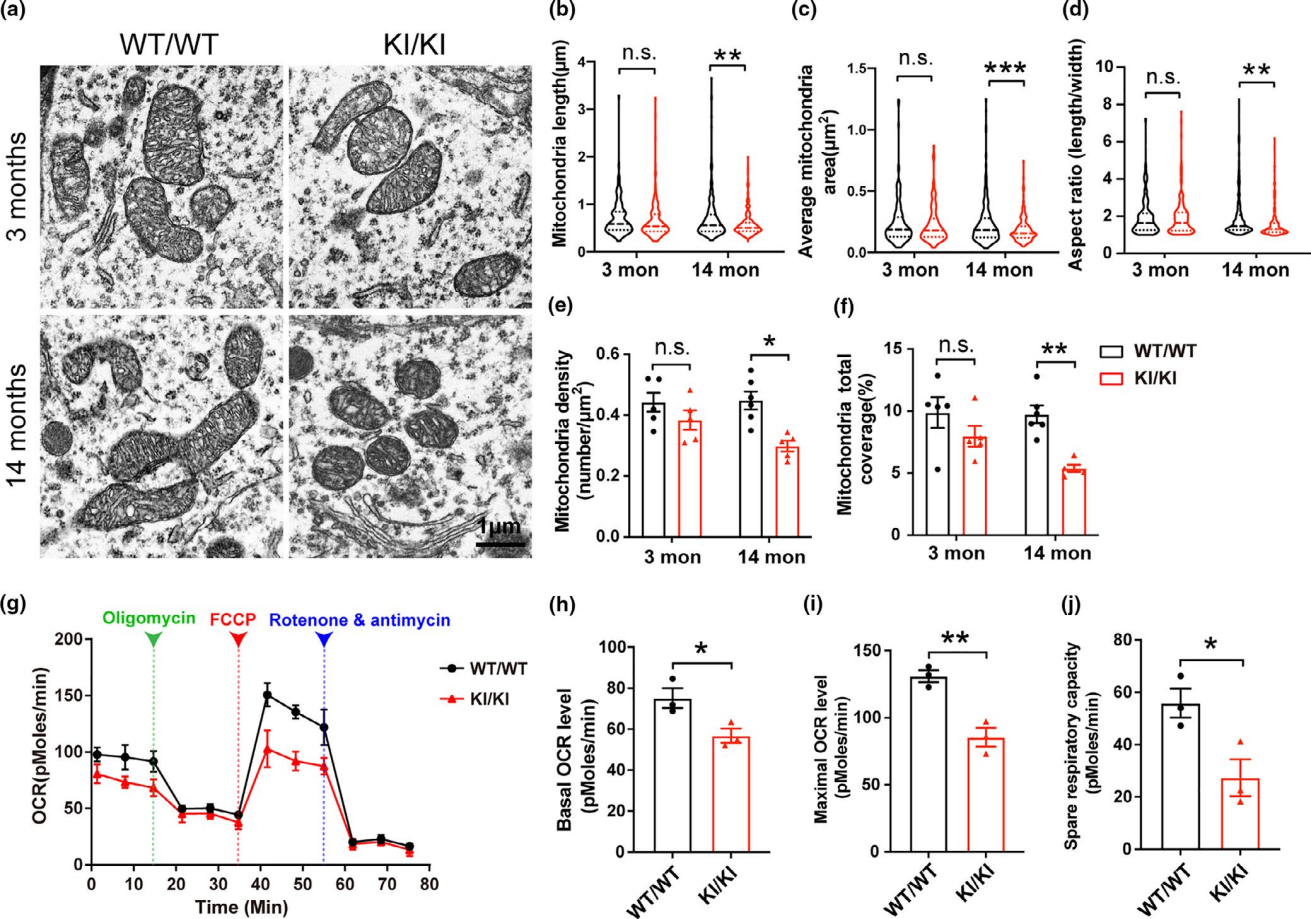
Mitochondrial morphology and respiratory deficits in *VPS35* D620N KI mice. (a) Representative electron microscopy picture from SNpc of *VPS35^D620N^* ^/^ *^D620N^* mice and WT controls at different ages as indicated. Scale bar, 1 μm. (b–f) Quantification of mitochondrial length (b), average mitochondria area (c), aspect ratio (d), mitochondrial density (e), and percentage coverage of cytoplasm by mitochondria (f), (200–350 mitochondria from 5 to 6 neurons per group; (b–d) were showed in violin plot; two‐way ANOVA with Tukey's post hoc test; mean ± *SEM*; *p < 0.05, **p < 0.01, ***p < 0.001, n.s., not significant). (g) Respiratory activity of synaptic mitochondria freshly isolated from cortex of 15‐month‐old *VPS35^D620N^* ^/^ *^D620N^* mice and control mice was analyzed by Seahorse XFe96 Assay. (h–j) Quantification of basal OCR (h), maximal OCR (i), and spare OCR (j) (Data are means ± *SEM* of 3 mice; unpaired *t* test; *p < 0.05, **p < 0.01, n.s., not significant)

Mitochondrial dysfunction represents a critical pathogenic step during the course of PD and recent studies demonstrated significant deficits in mitochondrial dynamics and quality control in PD (Ammal Kaidery & Thomas, 2018). To determine the impact of *VPS35* D620N mutation on the morphology and structure of mitochondria in vivo, electron microscopy analysis of neurons in the SNpc from *VPS35^D620N^* ^/^ *^D620N^* and WT mice at 3 and 14 months of age was performed (Figure 4a). Mitochondria appeared normal in 3‐month‐old *VPS35^D620N^* ^/^ *^D620N^* mice compared with age‐matched WT control mice, but became significantly shorter, rounder, and smaller in 14‐month‐old *VPS35^D620N^* ^/^ *^D620N^* mice. Indeed, quantification analysis revealed significantly reduced mitochondrial length, size, and aspect ratio (mitochondria long axis/short axis) in 14‐month‐old *VPS35^D620N^* ^/^ *^D620N^* mice (two‐way ANOVA, *p* < 0.01) (Figure 4b–d), suggestive of mitochondrial fragmentation. There was a trend toward reduced mitochondrial density and somatic areas coverage of mitochondria in the 3‐month‐old *VPS35^D620N^* ^/^ *^D620N^* mice which both became significant in the 14‐month‐old *VPS35^D620N^* ^/^ *^D620N^* compared with their age‐matched controls (two‐way ANOVA, *p* = 0.012 and *p* = 0.0022, respectively) (Figure 4e,f).

To determine the impact of *VPS35* D620N mutation on mitochondrial function, mitochondrial respiration was measured by Seahorse XFe96 analyzer in cortical synaptic mitochondria freshly isolated from the brains of 15‐month‐old *VPS35^D620N^* ^/^ *^D620N^* mice and WT controls. Basal OCR, maximal OCR, and spare OCR were significantly reduced in 15‐month‐old *VPS35^D620N^* ^/^ *^D620N^* mice compared with control mice (unpaired *t* test, *p* < 0.05) (Figure 4g–j).

**FIGURE 5:**
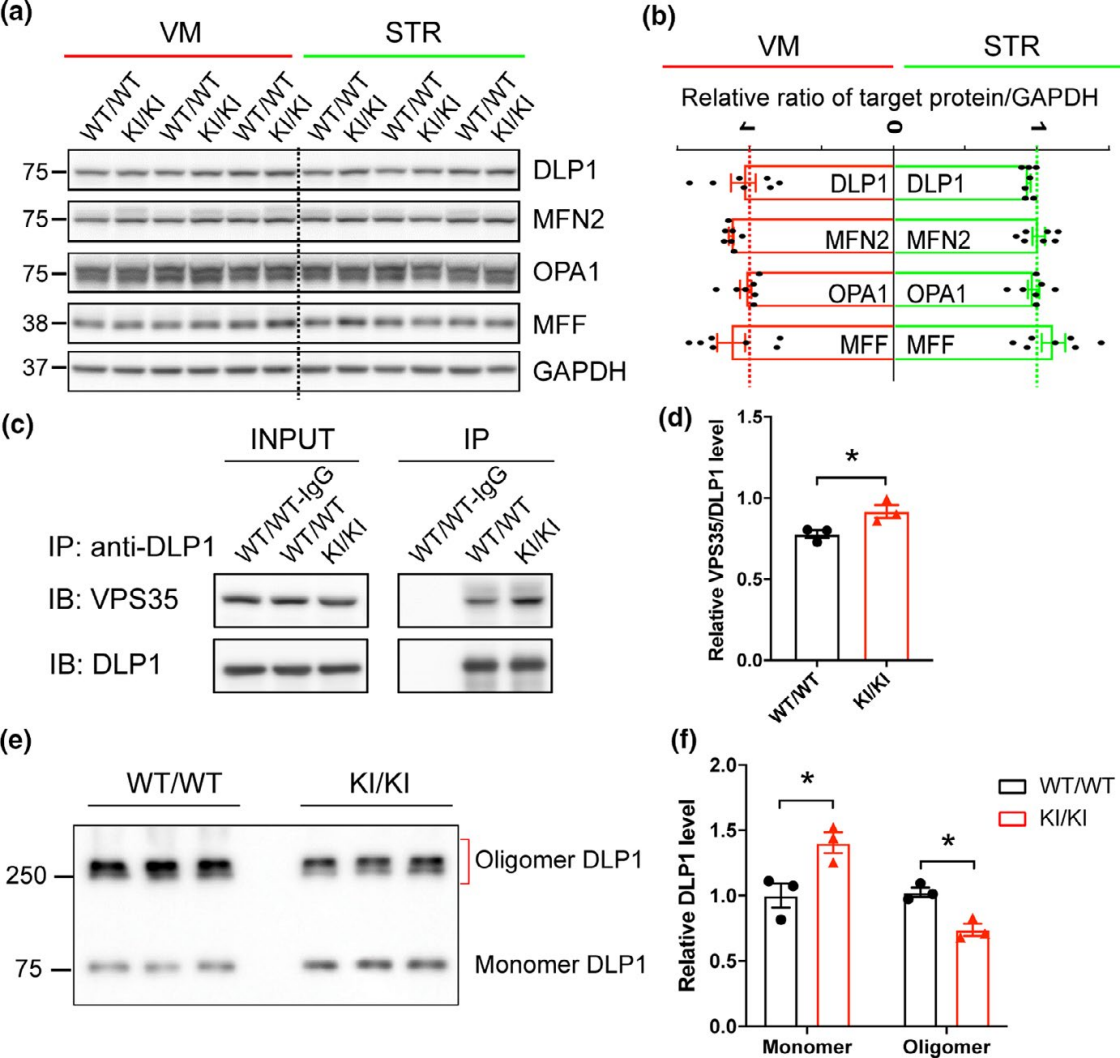
Changes in mitochondrial fission/fusion proteins in *VPS35* D620N KI mice. (a–b) Representative Western blots (a) and quantification (b) of mitochondrial fission/fusion proteins including DLP1, MFN2, OPA1, and MFF in VM and STR extracts from *VPS35^D620N^* ^/^ *^D620N^* and WT controls at 15‐month‐old (*n* = 7; Student's *t* test, unpaired, two‐tailed; data are shown as mean ± *SEM*; n.s., not significant). (c–d) Representative Western blot analysis (c) and quantification (d) of VPS35 in DLP1 immunoprecipitates from brain homogenates of *VPS35^D620N^* ^/^ *^D620N^* and WT mice (*n* = 3; Student's *t* test, unpaired, two‐tailed; data are shown as mean ± *SEM*; *p < 0.05). (e–f) Representative Western blot of DLP1 (e) and quantification (f) of oligomeric and monomeric DLP1 in the DSS‐treated mitochondrial fraction of VM from *VPS35^D620N^* ^/^ *^D620N^* and WT mice at 15‐month‐old (*n* = 3; unpaired *t* test; *p < 0.05)

To explore how *VPS35* D620N mutation causes extensive mitochondrial fragmentation and dysfunction, we investigated proteins involved in the regulation of mitochondria fission/fusion including DLP1, MFN2, OPA1, and MFF and found no changes in their expression in VM or STR extracts between *VPS35^D620N^* ^/^ *^D620N^* mice and WT controls at 15 months of age (Figure 5a,b). It was previously demonstrated that *VPS35* D620N mutant increased interaction with DLP1 in vitro which led to increased mitochondrial fission through efficient turnover of the mitochondrial DLP1 complexes (Wang et al., 2016). We confirmed increased VPS35‐DLP1 interaction in vivo in the whole brain homogenates from VM of *VPS35^D620N^* ^/^ *^D620N^* mice compared with that of WT control mice by co‐immunoprecipitation analysis (unpaired *t* test, *p* = 0.04) (Figure 5c,d). To determine the levels of mitochondrial DLP1 complexes in vivo, we prepared mitochondrial fractions from VM of 15‐month‐old *VPS35^D620N^* ^/^ *^D620N^* mice and age‐matched WT controls and exposed mitochondrial fraction to the irreversible crosslinking agent DSS to allow for detection of both oligomeric and monomeric form of DLP1 in mitochondria by Western blot after ultracentrifugation. Levels of mitochondrial oligomeric DLP1 complexes were significantly decreased in *VPS35^D620N^* ^/^ *^D620N^* mice compared with age‐matched WT controls (unpaired *t* test, *p* = 0.014) (Figure 5e,f). In contrast, levels of mitochondrial monomeric DLP1 were significantly increased in *VPS35^D620N^* ^/^ *^D620N^* mice (unpaired *t* test, *p* = 0.031) (Figure 5e,f).

### 2.6 Increased vulnerability of *VPS35* D620N KI mice to MPTP exposure

**FIGURE 6:**
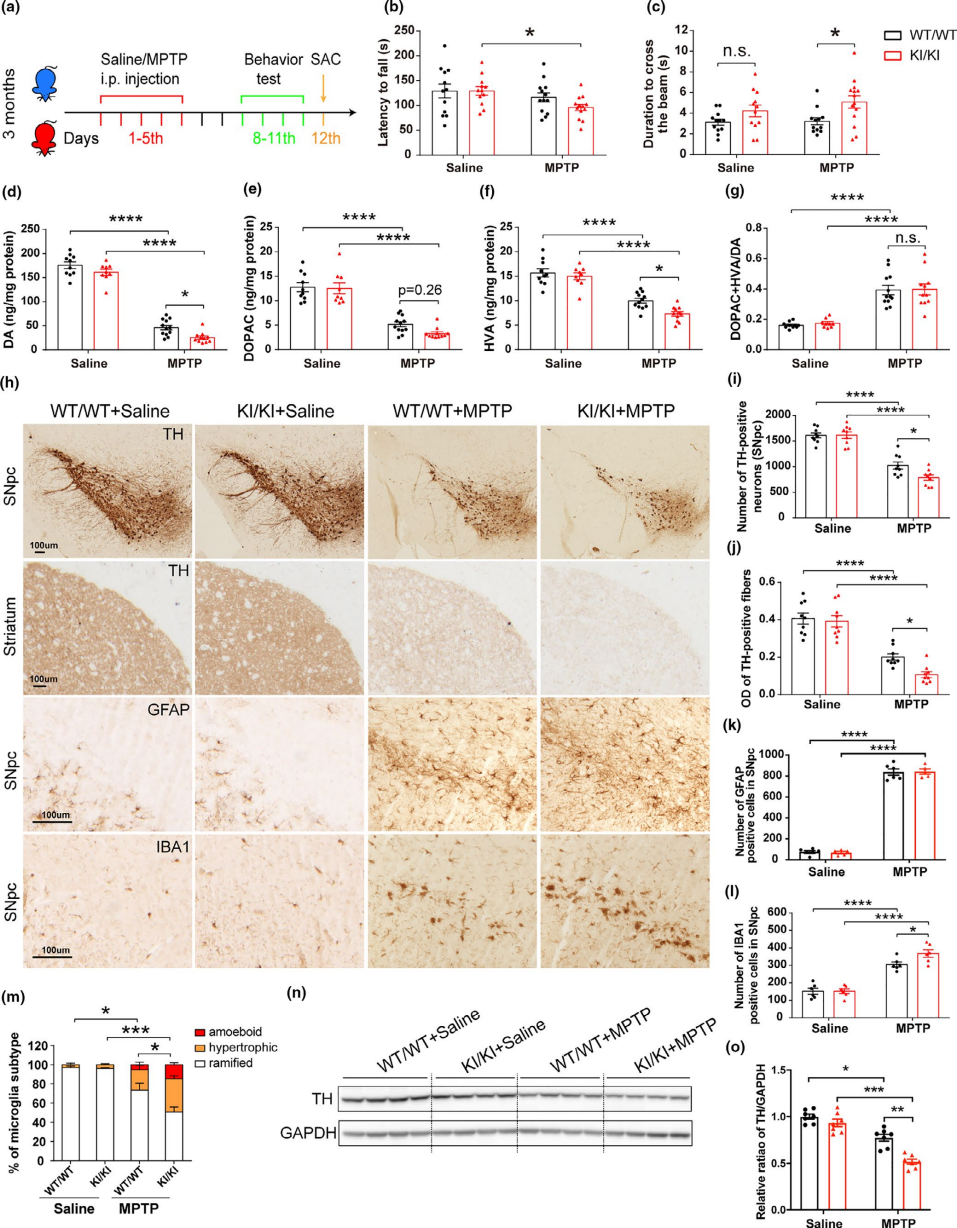
Increased MPTP vulnerability of *VPS35* D620N KI mice. (a) A schematic diagram depicts the experimental design. (b–c) Exacerbated motor function deficits in *VPS35^D620N^* ^/^ *^D620N^* mice as measured by the latency to fall in rotarod test (b) and duration to cross the narrow beam (9 mm) in beam walking test (c) (*n* = 12–14/group). (d–g) Concentrations of DA (d), DOPAC (e), HVA (f), and DA turnover rate (DOPAC+HVA/DA) (g) in the STR of *VPS35^D620N^* ^/^ *^D620N^* and WT controls injected with MPTP/Saline as indicated (*n* = 10–12/group). (h) Representative images of TH, GFAP, and IBA1 immunostaining in SNpc and STR from *VPS35^D620N^* ^/^ *^D620N^* and WT mice injected with MPTP/Saline as indicated. Scale bar, 100 μm. Quantification of TH‐positive neurons in SNpc (i), OD of TH‐positive fibers in STR (j), GFAP‐positive cells (k) and IBA1‐positive cells (l,m) in SNpc (*n* = 9/group). Representative Western blots (n) and quantification (o) of TH in VM extracts from *VPS35^D620N^* ^/^ *^D620N^* mice and WT controls injected with MPTP/Saline as indicated (*n* = 7/group). Two‐way ANOVA with Tukey's post hoc test; mean ± *SEM*; *p < 0.05, **p < 0.01, ***p < 0.001, n.s., not significant

*VPS35* D620N mutation was also found in sporadic PD patients (Ando et al., 2012). To investigate whether D620N mutation impacts the vulnerability to PD‐related environmental factors, 3‐month‐old *VPS35^D620N^* ^/^ *^D620N^* mice and their WT littermates were exposed to the neurotoxin MPTP. A schematic diagram of the experimental design was shown in Figure 6a. Behavioral tests including rotarod, beam walking, open field, and grip strength were carried out before mice were sacrificed. No differences were found in body weight between these two genotypes before or after injection (not shown). While this MPTP treatment regimen did not cause significant motor function deficits in WT mice compared with saline‐treated WT mice as measured by either rotarod test or beam walking test, it caused significant decrease in the latency to fall in the rotarod test in *VPS35^D620N^* ^/^ *^D620N^* mice compared with saline‐treated *VPS35^D620N^* ^/^ *^D620N^* mice (two‐way ANOVA, *p* = 0.041) (Figure 6b). *VPS35^D620N^* ^/^ *^D620N^* mice needed significantly longer time to cross the 9 mm beam compared with MPTP‐treat WT mice (two‐way ANOVA, *p* = 0.045) (Figure 6c). Neither *VPS35* D620N mutation nor MPTP treatment causes motor deficits concerning wide‐beam (16 mm) walking test, open field test, and grip strength test (Figure S5a‐i). As expected, MPTP induced a significant reduction in the DA, DOPAC, and HVA levels in both WT and *VPS35^D620N^* ^/^ *^D620N^* mice (two‐way ANOVA, *p* < 0.0001) (Figure 6d–g). Notably, *VPS35^D620N^* ^/^ *^D620N^* mice injected with MPTP showed a significantly greater reduction in DA and HVA levels compared with WT controls two‐way ANOVA, *p* = 0.036 and *p* = 0.04, respectively) (Figure 6d,f).

No difference in the number of TH‐positive neurons in SNpc or optical density of TH‐positive fibers in STR were found between saline‐treated *VPS35^D620N^* ^/^ *^D620N^* and WT mice (Figure 6h–j). MPTP treatment caused significant reduction in TH‐positive neurons in WT mice (~36%) which became significantly exacerbated in *VPS35^D620N^* ^/^ *^D620N^* mice (~51%) (two‐way ANOVA, *p* = 0.036) (Figure 6h,i). Similarly, MPTP‐induced reduction in striatal TH‐positive fibers was significantly exacerbated in *VPS35^D620N^* ^/^ *^D620N^* mice compared with that of WT mice (two‐way ANOVA, *p* = 0.018) (Figure 6h,j). MPTP treatment induced significant astrogliosis (GFAP staining) in SNpc or STR of both WT and *VPS35^D620N^* ^/^ *^D620N^* mice although not much difference was found between the two genotypes (Figure 6h,k; Figure S6a,b). MPTP significantly increased microgliosis (IBA1 staining) in SNpc of WT mice which became exacerbated in *VPS35^D620N^* ^/^ *^D620N^* mice (two‐way ANOVA, *p* = 0.038) (Figure 6h,l). Activated microglia (hypertrophic and amoeboid microglia) were obviously increased in response to MPTP in *VPS35^D620N^* ^/^ *^D620N^* mice (Figure 6h,m). However, neither *VPS35* D620N mutation nor MPTP treatment induced microgliosis in STR (Figure S6a,c). Western blot and quantification analysis showed greater reduction of TH expression levels in VM extracts from MPTP‐treated VPS35D620N/D620N mice compared with MPTP‐treated WT controls (two‐way ANOVA, *p* = 0.008) (Figure 6n,o).

## 3 DISCUSSION

In this study, we found that endogenous expression of *VPS35* D620N at physiological level is sufficient to induce a spectrum of characteristic features of PD in the *VPS35* D620N KI mouse model in an age‐dependent manner. These included mild and progressive motor deficits, significant changes in the levels of DA and DA metabolites in the STR, and robust neurodegeneration of the DA neurons in the SNpc and DA terminals in the STR, accompanied by increased neuroinflammation, and accumulation and aggregation of α‐syn in DA neurons in 15‐ to 16‐month‐old D620N KI mice. In addition, endogenous expression of *VPS35* D620N mutant caused mitochondrial fragmentation and impaired respiration of synaptic mitochondria in the brain, confirming the involvement of abnormal mitochondrial dynamics in vivo. Mechanistically, we found *VPS35* D620N mutation increased VPS35‐DLP1 interaction and enhanced removal of mitochondrial DLP1 complexes in vivo which likely caused fragmented mitochondria in the brain through enhanced fission. Finally, MPTP treatment resulted in greater motor deficits and degeneration of the nigrostriatal DA pathway in *VPS35^D620N^* ^/^ *^D620N^* mice, indicating that *VPS35* D620N mutation increased vulnerability of DA neurons to environmental toxins.

Development of animal models that faithfully recapitulate characteristic features of PD is necessary and critical for the field, but alteration of a single PD‐related gene in mouse models to recapitulate the full spectrum of PD symptoms proves to be difficult. While several transgenic models for α‐syn and LRRK2 mutations demonstrated robust DA neurodegeneration, they usually involved overexpression of the mutant (Karim et al., 2020; Yue, 2012). KI mouse models, however, allow studies of disease‐associated mutants at physiological expression patterns and levels in vivo representing more accurate disease models. Recent studies on young mice of two different D620N KI mouse models found no overt motor deficits or pathological changes in young mice, but reported alterations [increases (Cataldi et al., 2018) or decreases (Ishizu et al., 2016)] in dopamine release, suggesting an early dysfunction in dopaminergic neurons in D620N KI mice. One of these studies on the same D620N KI mice used in the current study reported increased dopamine turnover in D620N KI mice at 3 month of age (Cataldi et al., 2018) which was not confirmed in our study either in 3‐month or 15‐ to 16‐month‐old mice. Our detailed characterization of the D620N KI mice along aging confirmed PD‐related pathological and motor deficits in 15–16 months but not in younger KI mice. Moreover, we found 3‐month‐old D620N KI mice were more vulnerable to MPTP‐induced neuronal loss and motor deficits. Collectively, these two latter studies demonstrated that early dopaminergic neuronal dysfunction caused by D620N mutation reported in the earlier studies (Cataldi et al., 2018; Ishizu et al., 2016) likely continues to evolve over time and/or accelerates following environmental insult which eventually leads to neurodegeneration in vivo.

Most recently, Chen et al., (2019) characterized another D620N KI mouse model derived from the crossing of the same conditional D620N KI mice (Jaxon Lab stock# 021807) with a different Cre‐delete line (e.g., CMV‐Cre transgenic mice). They were the first to report a significant progressive reduction in DA neurons in SNpc at 13 month of age (Chen et al., 2019). However, other than the overt loss of dopaminergic neurons in the SNpc, there were multiple differences in D620N mutation caused deficits between our current model and this related model (Chen et al., 2019): (1) Chen et al., (2019) found more than 30% dopaminergic neuronal loss in both the heterozygous and homozygous D620N KI mice, supporting a gain‐of‐toxic function of the D620N mutation. In contrast, our study demonstrated around 12% loss of dopaminergic neurons only in the homozygous but not in the heterozygous *VPS35^D620N^* ^/^ *^WT^* mice, indicating a gene dosage effect, which appears to support a loss‐of‐function mechanism underlying neurodegeneration. In this regard, it is known that VPS35 plays a critical role in the maintenance and survival of DA neurons and VPS35 depletion caused loss of DA neurons in mice (Tang, Liu, et al., 2015); (2) Despite the overt loss of substantia dopaminergic neurons and extensive axonal damage, Chen et al. surprisingly found no reduction in the density of TH‐positive dopaminergic nerve terminals in the STR of D620N KI mice which may be due to a compensatory resprouting of the remaining dopaminergic axonal processes. However, we found around 25% loss of TH‐positive dopaminergic axonal terminals in the STR in our model, greater than the loss of SNpc dopaminergic neurons, supporting a dying back mode. Indeed, dystrophic myelinated axons were frequently found in dopaminergic neurons in the 14‐month D620N KI mice in the current study; (3) Chen et al., (2019) found no significant changes to aspects of motor function or DA levels in the STR at 3 and 13 months of age (Chen et al., 2019). In contrast, our D620N KI mice demonstrated both age‐dependent motor functional deficits and significant reduction in levels of DA and its metabolites in the STR; and (4) increased tau but no α‐syn pathology was identified in Chen's model (Chen et al., 2019). However, we did not find any tau pathology stained by either AT8 or MC‐1. Instead, we found accumulation and aggregation of α‐syn in the DA neurons and extensive neuroinflammation in the D620N KI mice. While it remains to be determined what caused different phenotypes among different D620N KI mouse models, subtle differences in the genetic background, rederivation procedures, and experimental environment might contribute. Overall, it appears that the constitutive homozygous D620N KI mice described in the present study successfully recapitulate many cardinal features of PD including neurodegeneration, neurochemical deficits, and motor deficits which makes it a more complete and faithful model of PD and thus provides a valuable tool for disease modeling and pharmaceutical studies.

Cytoplasmic inclusions containing α‐syn aggregates, referred to as Lewy bodies, are the signature neuropathological hallmarks of PD (Spillantini et al., 1997). While PD patients bearing *VPS35* D620N mutation resemble typical idiopathic PD patients clinically, only one patient carrying single *VPS35* D620N mutation has been evaluated at autopsy thus far which unfortunately lacked an assessment of substantia nigra (Wider et al., 2008). A most recent report of a patient with a rare VPS35 mutation and a FBXO7 mutation displayed classic Lewy body pathology in the substantia nigra and numerous dystrophic neurites, dots, and neuronal granular cytoplasmic positivity of α‐syn immunoreactivity were identified in multiple brain regions (Mensikova et al., 2019), implicating that VPS35 mutation, in combination of other factors, could impact α‐syn pathology in PD patients. In this regard, several mechanistic studies have suggested a functional interaction of VPS35 and α‐syn: *VPS35* D620N mutant impaired endosome‐to‐TGN trafficking of CI‐M6PR which led to missorting and dysfunction of cathepsin D, a lysosome protease responsible for degradation of α‐syn (Follett et al., 2014). Tang, Erion, et al. (2015) found virus‐mediated overexpression of *VPS35* D620N mutation in mice cause accumulation and aggregation of α‐syn in midbrain through impaired endosome‐to‐Golgi retrieval of Lamp2a, which is a receptor of chaperone‐mediated autophagy that is important for lysosomal clearance of α‐syn (Tang, Erion, et al., 2015). *VPS35* D620N mutant also facilitates intracellular seeding of pathogenic α‐syn between neurons (Dhungel et al., 2015). Indeed, we found accumulation of somatic α‐syn in DA neurons in the SNpc of *VPS35* D620N KI mice at advanced age. More importantly, the accumulation of α‐syn is accompanied by increased soluble α‐syn oligomers and insoluble α‐syn HMW bands in VM extracts from *VPS35* D620N KI mice. These data demonstrated that *VPS35* D620N mutation promotes accumulation and aggregation of α‐syn in vivo for the first time and thus support the notion that VPS35 mutation likely promotes the development of α‐syn pathology in PD. Our initial study found significantly reduced levels of Lamp2a along with increased number of lysosomes, especially those lysosomes with dense body inclusions, in the *VPS35* D620N KI mice which suggested that impaired autophagy likely contributes to VPS35‐mediated development of α‐syn pathology in vivo.

One important finding of present study is that endogenous levels of *VPS35* D620N mutant caused mitochondrial fragmentation and mitochondrial respiratory dysfunction in substantia nigra at advanced age. It should be noted that there were trends toward abnormal mitochondrial dynamics in dopaminergic neurons in younger D620N KI mice which could be the basis of early dysfunction of these dopaminergic neurons (Cataldi et al., 2018; Ishizu et al., 2016). These results lend strong support of the crucial role of impaired mitochondrial dynamics in the pathogenesis of VPS35 mutation‐associated PD. It is consistent with prior studies demonstrating *VPS35* D620N mutation induces mitochondrial fragmentation and cell death in vitro and in mouse substantia nigra after viral‐mediated overexpression (Wang et al., 2016). In fact, expanding evidence shows that either exposure to PD‐related neurotoxins or alterations of many of the PD genetic factors including α‐syn, LRRK2, DJ‐1, PINK1, and Parkin cause abnormal mitochondrial dynamics and mitochondrial dysfunction, and inhibition of DLP1‐dependent mitochondrial fission or promotion of mitochondrial fusion alleviated DA neuronal death both in vitro and in vivo (Deng et al., 2008; Kamp et al., 2010; Liu et al., 2019; Nakamura et al., 2011; Rappold et al., 2014; Wang, Petrie, et al., 2012; Wang, Yan, et al., 2012). Together, these data support an important role of abnormal mitochondrial dynamics in both sporadic and familial forms of PD which may be pursued as a potential therapeutic target. Mechanistically, we found *VPS35* D620N mutation increased VPS35‐DLP1 interaction and enhanced turnover of mitochondrial DLP1 complexes in vivo, which lends strong support of the notion that VPS35 promotes mitochondrial fission through efficient turnover of mitochondrial DLP1 complexes after fission (Wang et al., 2016). However, given the functional interactions between VPS35 and α‐syn and LRRK2 (Dhungel et al., 2015; Mir et al., 2018), *VPS35* D620N may also exert damaging effects through LRRK2 and/or α‐syn.

Parkinson's disease is a multifactorial disorder in which both genetic and environmental risk factors are involved and *VPS35* D620N mutation is found in both familial and sporadic PD patients. MPTP is a well characterized neurotoxin that results in pathology and phenotype similar to PD both in human and rodents (Smeyne & Jackson‐Lewis, 2005). In the current study, although 3‐month‐old *VPS35^D620N^* ^/^ *^D620N^* mice appear indistinguishable from WT control mice at basal condition, they displayed significantly greater DA neuron loss and exacerbated deficits in motor functions and reduction in DA and its metabolites after exposure to MPTP compared with that of WT control mice. The D620N mutation also exacerbates the microgliosis as showed by increased microglial numbers and active morphology change. These data clearly demonstrated that *VPS35* D620N mutation increased the vulnerability of the nigrostriatal system to PD‐related neurotoxin which thus supports the notion that genetic factors and environmental factors contribute to aggregate and advance disease pathogenesis in PD. MPTP is specifically taken up by DA neurons and metabolized to MPP^+^ which inhibits mitochondrial complex I and causes DA neuronal death (Smeyne & Jackson‐Lewis, 2005). Recent data suggest that mitochondrial fragmentation and impaired autophagy were also involved in MPP^+^‐induced neuronal death (Wang et al., 2011; Zhu et al., 2007). Since *VPS35* D620N mutation also impaired mitochondrial dynamics and autophagy, it would be of interest to investigate the synergistic effects of these deficits in mediating greater DA loss induced by the combination of these factors.

## 4 CONCLUSION

Collectively, we find homozygous *VPS35* D620N KI mice recapitulate cardinal features of PD which represents a more faithful PD mouse model. It provides a unique opportunity to unravel critical pathogenic roles of *VPS35* D620N mutation and pharmacology studies in PD without the artificial overexpression and random integration of a transgene. Our data support the involvement of VPS35 in the development of α‐syn pathology in vivo. We also demonstrated endogenous levels of *VPS35* D620N mutant cause mitochondrial fragmentation and dysfunction in vivo, supporting the crucial role of mitochondrial dynamics in PD pathogenesis.

## 5 EXPERIMENTAL PROCEDURES

### 5.1 Animals and treatment

Constitutive *VPS35* D620N KI mice were obtained from The Jackson Labs (B6(Cg) ‐ Vps35^tm1.1Mjff^/J; Stock No: 023409). Mice were housed under standard conditions, and all animal studies were approved by the Institutional Animal Care and Use Committee (IACUC) of Case Western Reserve University. PCR of genomic DNA was used for genotyping offspring at 3 weeks and was reconfirmed after sacrifice. Body weights of mice from 1 to 15 months were measured every 2 weeks. Mice were sacrificed by cervical dislocation after anesthesia at different ages (6‐, 10‐ and 14‐ to 16‐month old). For MPTP treatment, 3‐month‐old *VPS35^D620N^* ^/^ *^D620N^* mice and their wild‐type (WT) littermates received intraperitoneal (i.p.) injection of either MPTP /HCl (sc‐206178, Santa Cruz; 25 mg/kg of body weight) or an equivalent volume of 0.9% saline for 5 consecutive days. All mice were sacrificed at 7 days post‐MPTP treatment. Numbers of mice used for each experiment is noted in legends.

### 5.2 Behavioral analysis

Both motor and non‐motor assessments were conducted with *VPS35* D620N KI mice and their WT littermates (*n* ≥ 12 mice per group, sex‐matched) at different ages. Mice were transferred to the testing room to acclimate for at least 1 h before behavior tests.

*Open* *field* *test* was used to assess general locomotor activity, anxiety, and aspiration to explore. Mice were placed in the center of a 50 × 50 cm arena and explored for 10 min under dim light. Videos were recorded and analyzed with ANY‐maze behavioral tracking software (Stoelting Co.). *Rotarod* *test* was carried out to evaluate motor coordination and balance using Pan lab Rota‐Rods (Harvard Apparatus). Mice were trained to stay on the rotarod bar for 6 trials over 3 days. For each trial, the rotation was set at a constant speed, but higher from trial to trial. During the test, the rod was set to accelerate from 4 to 40 rpm over 10 min. The latency to fall from the rod and the maximum speed reached was recorded. *Beam* *walking* *test* was used to assess fine motor coordination and balance. The beam apparatus consists of 60 cm wooden beams with a flat surface of 9 mm width resting 50 cm above the table top on two poles. Mice were allowed to traverse the beams with a stimulus (60 W light) placed near the start and an enclosed safety platform at the end. Mice were initially trained to cross the beam for 6 trials over 3 days before being tested in two consecutive trials on 9 mm beams. The time taken to cross the center 40 cm of the beam was recorded. *Grip* *strength* *test* was used to measure muscle force of forelimbs and hindlimbs using the grip strength test meter from Bioseb. The mouse forepaws or hind paws were placed on a metal pull bar before they were pulled horizontally away from the bar by the tail until their grip was released. Each mouse was tested 5 times, and the maximum force was recorded. *Buried* *pellet* *test* was used to evaluate general olfactory function (Lehmkuhl et al., 2014). Mice were food‐restricted with 0.2 g chow/day from 2 days prior to and during the test. Body weight was maintained at ~90% of the original body weight. Each mouse received one trial per day over 5 consecutive days. In the clean test cage, a 1‐g food pellet was buried 0.5 cm below the surface of a 3‐cm‐deep layer of mouse bedding material. During test, mouse was first placed in the center of the test cage. Of note, the location of the food pellet was randomly changed daily. The latency to uncover and eat the pellet was recorded. *One*‐*hour* *stool* *collection* was tested to screen for constipation in mice (Taylor et al., 2010). Each test mouse was placed in a clean cage and monitored for 1 h. Fecal pellets were collected into sealed 1.5‐ml tubes immediately after expulsion to avoid evaporation. Tubes were then weighed to obtain the wet weight of the stool. After that, tubes were dried overnight at 65°C and reweighed to get the dry weight.

### 5.3 Immunohistochemistry, confocal immunofluorescence, and electron microscopy

Immunohistochemistry was performed by the peroxidase anti‐peroxidase protocol as described before (Zhao et al., 2017). To quantify the number of DA neurons in SNpc, formalin‐fixed paraffin‐embedded mouse brains were sliced into 6‐µm thickness coronal sections. SN was delineated from 2.9 to 4.1 mm posterior to the bregma; thus, the sections were cut from about 2.5 to 4.5 mm posterior to the bregma to ensure the entire SN area was included. Sections were immersed in 2 changes of xylene, followed by descending series of ethanol (100%–95%–70%–50%), and finally into Tris‐buffered saline (TBS: 50 mM Tris, 150 mM NaCl, pH = 7.6). Then, sections were put into citrate buffer and went through pressure cooking (Biocare) for antigen retrieval. After blocking for 30 min in 10% normal goat serum, sections were incubated with primary antibodies overnight at 4°C. Species specific secondary antibodies and PAP complexes were then applied at room temperature. After 3 washes of Tris buffer, the sections were developed with DAB (Dako) and dehydrated in 70%–95%–100% ethanol and xylene. Sections were then coverslipped with Permount. Every other 3 sections were stained with tyrosine hydroxylase (TH) antibody across the midbrain. DA neurons on each section were quantified using NIH ImageJ and then stacked together as the amount of DA neurons in SN area. DA innervation was assessed by the optical density (OD) of TH‐positive fibers from 6 coronal sections spanning the STR and was quantified using Axiovision software. All images were acquired on an Axiophot with an Axiocam (Zeiss). For immunofluorescence, mice brain sections were stained as previously described (Wang et al., 2016). Confocal images were collected by Leica STED SP8 system. For electron microscopy analysis, brain samples were collected and fixed as previously described (Wang et al., 2017). Brain slices of about 1 mm thick were made, and SNpc region was sampled and embedded in Epon. Sections were then sequentially stained with 2% acidified uranyl acetate followed by Sato's triple lead staining and examined by FEI Tecnai T12 electron microscope. SNpc neurons were imaged by microscopist without prior knowledge of age and genotype. Indicators of mitochondria were measured by NIH ImageJ software. Antibodies used were directed against anti‐TH (Millipore, MAB318), anti‐GFAP (Invitrogen, PA5‐16291), anti‐Iba1 (Invitrogen, PA5‐21274), and anti‐alpha synuclein (Abcam, Ab1903).

### 5.4 Detergent‐solubility fractionation of α‐synuclein

Triton X‐100 was added to the VM lysates of mice to make a final concentration of 1%. Samples were then incubated on ice for 30 min followed by centrifugation (18,000 *g*, 30 min, 4°C) (Klucken et al., 2004). The supernatant was saved as Triton X‐100 soluble fraction. The pellet was re‐dissolved in 2% SDS‐sample buffer and sonicated for 10 s, and saved as Triton X‐100 insoluble fraction. Equal amounts of samples were then loaded onto 10% Tris/glycine gels for Western blot analysis.

### 5.5 Western blot and immunoprecipitation

Dissected VM and STR were individually homogenized in RIPA buffer (Abcam) with added protease and phosphatase inhibitors (Roche). Tissue homogenates were centrifuged at 18,000 *g* for 20 min, and the supernatants were quantified by BCA assay (Pierce). Equal amounts of protein extract (20–30 μg) were resolved with SDS‐PAGE and loaded onto 8%–12% Tris/glycine gels for running. Proteins were transferred to PVDF membrane (0.45 μm) at constant 30 volts overnight (4°C). Membranes were then blocked with 10% non‐fat milk. For detection of alpha synuclein, membranes were incubated with 0.04% PFA (in PBS) for 15 min before blocking. Membranes were incubated with primary antibodies for 2 h at RT or overnight at 4°C. Blots were washed in TBST (5 min × 3 times). Secondary antibodies were then applied, and the blots were washed and developed with the Immobilon western chemiluminescent horseradish peroxidase substrate (Millipore). Immunoprecipitation was performed with DLP1 antibody‐coated magnetic beads in 1% NP40 buffer using the Dynabeads Protein G‐IP Kit (Invitrogen) as previously described (Wang et al., 2016). Primary antibodies used include anti‐VPS35 (Abcam, a157220), anti‐VPS26 (Abcam, a23892), anti‐VPS29 (Abcam, ab236796), Synaptophysin (Invitrogen, MA5‐11475), PSD 95 (cell signal, 3409), VMAT2 (Santa Cruz, sc15314), DAT (Santa Cruz, sc32259), alpha synuclein (Abcam 1903), Mff (ProteinTech, 17090–1‐AP), Mfn2 (Abcam, 56889), anti‐DLP1 (BD Biosciences, 611112), OPA1 (BD Biosciences, 12606), anti‐GAPDH (Cell Signal, 2118), and anti–β‐actin (Chemicon, MAB1501). Images were analyzed for band intensity with ImageJ software.

### 5.6 Measurement of biogenic amines by high performance liquid chromatography with electrochemical detection (HPLC‐ECD)

The brains of 14‐month‐old KI and WT mice were quickly removed and washed with ice‐cold phosphate‐buffered saline (PBS). The STR of half brain was removed. The tissue samples were homogenized and analyzed for DA and its metabolites (DOPAC and HVA) by HPLC via the Neurochemistry Core of the Vanderbilt University using an Antec Decade II (oxidation: 0.65) electrochemical detector operated at 33°C as described previously (Ward et al., 2018).

### 5.7 Mitochondrial oxygen consumption measurement in synaptosomes

The real‐time oxygen consumption rate (OCR) in synaptic mitochondria in synaptosomes was measured by the Seahorse XFe96 Analyzer as described before (Jiang et al., 2018). Briefly, cortex was rapidly removed and homogenized in glass homogenizer containing 1.5 ml ice‐cold ‘Sucrose Medium’ (320 mM sucrose, 1 mM EDTA, 0.25 mM dithiothreitol, pH 7.4). The homogenate was centrifuged at 1000 *g* for 10 min at 4°C. The supernatant was carefully layered on top of a discontinuous Percoll gradient (3 ml layers of 3%, 10% and 23% Percoll in Sucrose Medium) followed by centrifugation at 32,500 *g* for 20 min at 4°C. The band of synaptosomes between 10% and 23% Percoll was diluted into ‘Ionic Medium’ (20 mM HEPES, 10 mM D‐ Glucose, 1.2 mM Na_2_HPO_4_, 1 mM MgCl_2_, 5 mM NaHCO_3_ 4°C, 5 mM KCl, 140 mM NaCl, pH 7.4) followed by centrifugation at 15,000 *g* for 15 min at 4°C. The final synaptosome pellets were then resuspended in Ionic medium and aliquoted into a polyethyleneimine‐coated XFe96 cell culture microplate (5 µg protein/well) followed by centrifugation at 3400 *g* for 30 min at 4°C. The Ionic medium was replaced with ‘Incubation medium’ (3.5 mM KCl, 120 mM NaCl, 1.3 mM CaCl_2_, 0.4 mM KH_2_PO_4_, 1.2 mM Na_2_SO_4_, 2 mM MgSO_4_, 15 mM D‐glucose, 4 mg/ml BSA, 37°C). The cell culture microplate was incubated and loaded into the Seahorse 96XFe analyzer following the manufacturer's instructions. ATP synthase inhibitor oligomycin (2 µM), uncoupler FCCP (1 µM), and complex I inhibitors antimycin A (0.5 µM) and rotenone (0.5 µM) were injected to the well sequentially.

### 5.8 Mitochondria cross‐link and DLP1 complex assay

Crude mitochondria were extracted from VM as described (Wang et al., 2016). After wash with PBS, the fresh mitochondria were incubated with 1.25 mM disuccinimidyl suberate (DSS, from freshly prepared stocks in DMSO) for 30 min at room temperature. Stop solution (1 M Tris, pH 7.5) was then added to a final concentration of 10–20 mM and incubate for 15 min. After centrifugation at 10,000 *g* for 10 min at 4°C, pellets were lysed in lysis buffer and loaded onto SDS‐PAGE.

### 5.9 Statistical analysis

All statistical analyses were performed blindly with GraphPad prism (version 7) software. Student's *t* test or one‐way/two‐way ANOVA were used to compare group differences. Data were normally distributed with similar variance between the groups. Statistical significance was taken as two‐sided *p* < 0.05.

## CONFLICT OF INTEREST

The authors declare that they have no competing interests.

## AUTHOR CONTRIBUTIONS

MN designed and carried out experiments, analyzed results, generated figures and wrote the first draft; FZ, KB, SLS, and ST helped to collect data; HF helped with electron microscopy; WW and JL contributed to the conception of the project, design of the experiments and the interpretation of results, and provided feedback on the manuscript; XZ conceived and directed the project, design of the experiments, interpreted the results and wrote the manuscript. All authors read and approved the final manuscript.

## Supporting information

Fig S1‐S6

Fig S7

## Data Availability

The data sets supporting the conclusions of this article are included within the article and its Supplemental Figures.
