# Supplementary material for: VPS35 D620N knockin mice recapitulate cardinal features of Parkinson’s disease
Source: Aging Cell. 2021 Mar 21;20(5):e13347. doi: 10.1111/acel.13347 (PMC8135078; doi:10.1111/acel.13347)
Supplement: Supplementary file 1 — Fig S1‐S6 [file ACEL-20-e13347-s001.pdf]

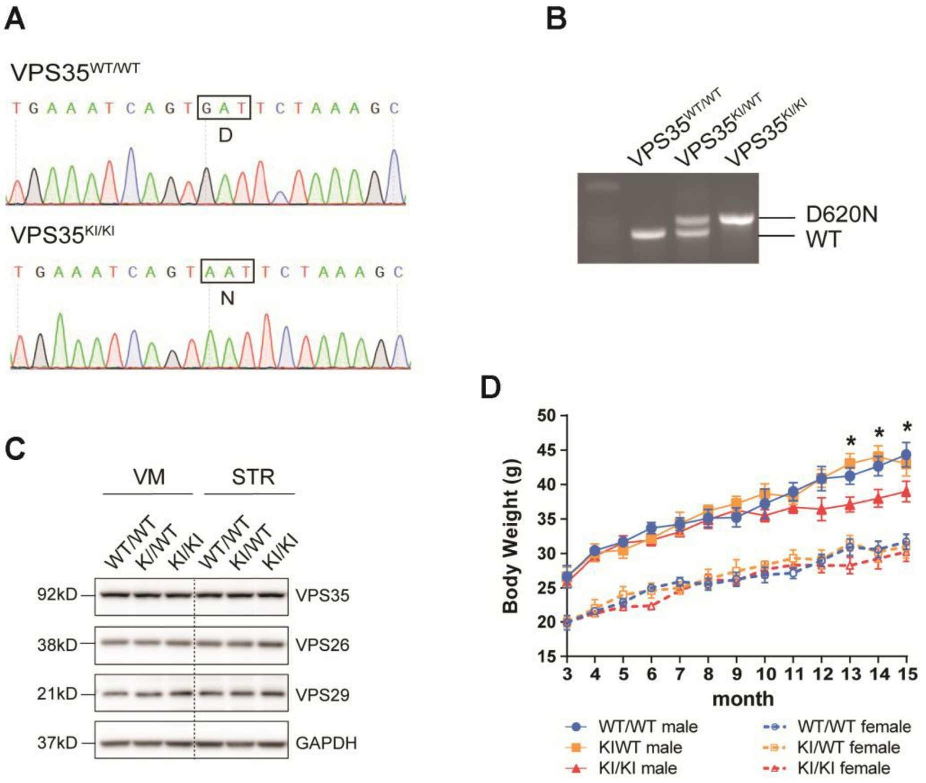

Supplemental Figure 1. Generation of VPS35 D620N KI mice. (A) Representative VPS35 genomic sequences showing the presence of the D620N mutation (GAT → AAT) in VPS35 D620N/D620N mice but not the VPS35 WT/WT mice. (B) Representative PCR genotyping of ear notch genomic DNA for VPS35 WT/WT, VPS35 D620N/WT and VPS35 D620N/D620N mice. (C) Representative Western blots of VPS35, VPS26 and VPS29 protein in ventral midbrain and striatum extracts from 14-mont-old mice of three genotypes (n=7/genotype). GAPDH was blotted as an internal loading control. (D) Body weight of mice from three genotypes (one-way ANOVA with Tukey's post hoc test). Data are shown as mean ± SEM; *P < 0.05.

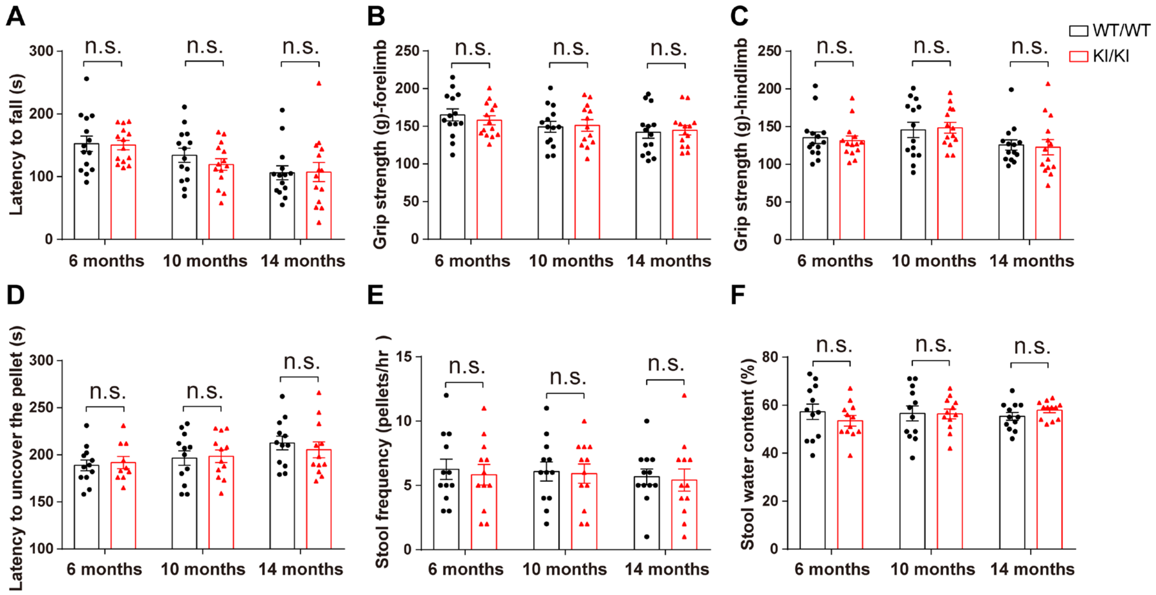

Figure S2 Motor and non-motor function analysis of $VPS35^{D620N/D620N}$ mice and littermate WT controls (6, 10, 14 months). (A) Latency to fall in rotarod test. (B) Forelimb grip strength. (C) Hindlimb grip strength. (D) Latency to uncover the pellet in buried pellet test. (E) Stool frequency in one-hour stool collection. (F) Stool water content in one-hour stool collection (n=13-14/group, two-way ANOVA with Tukey's post hoc test). Data are shown as mean ± SEM; n.s., not significant.

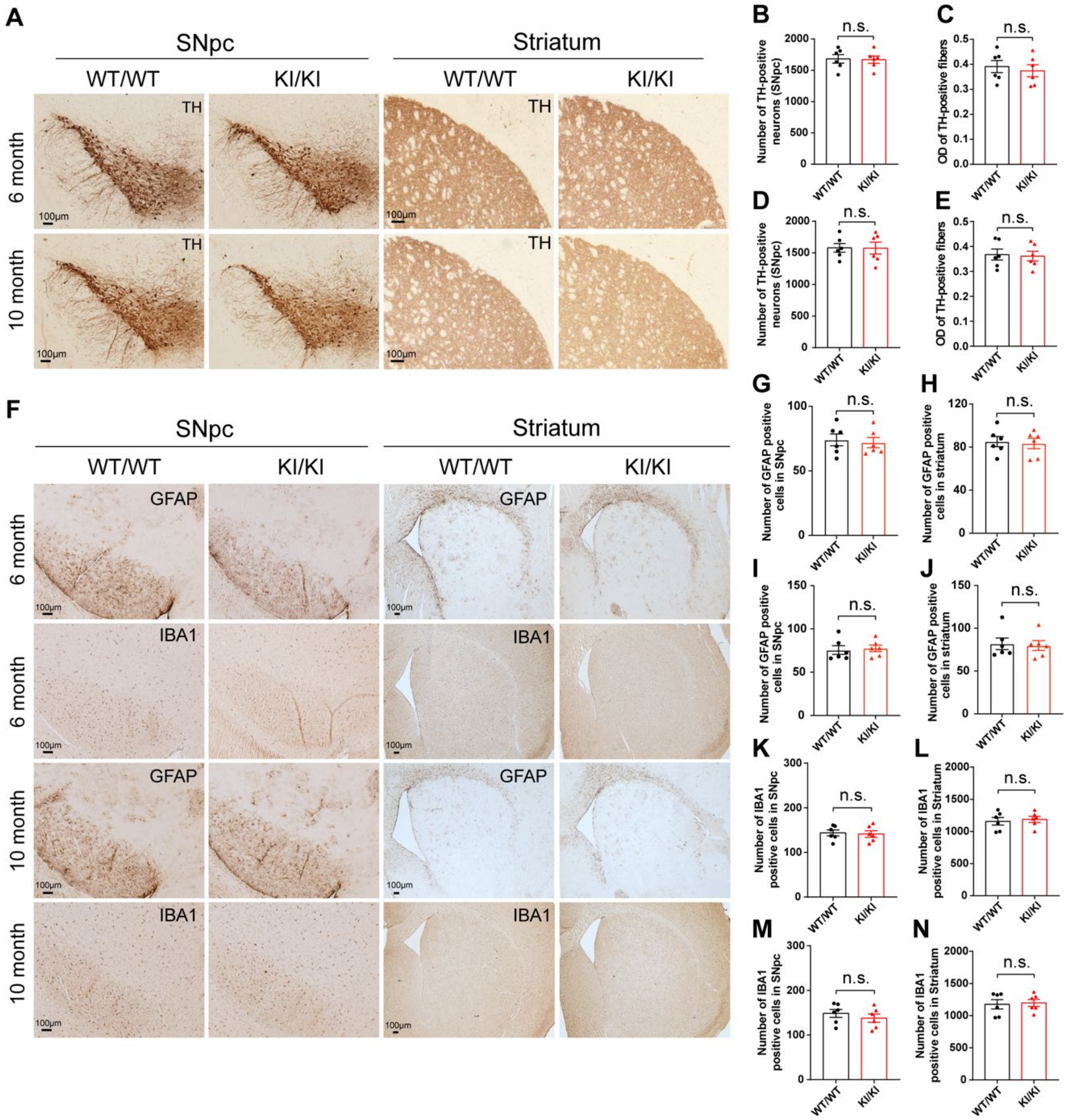

Figure S3. Lack of neuropathological changes in 6 and 10 months old homozygous *VPS35* D620N KI mice. (A) Representative images of TH immunostaining in SNpc and striatum of $VPS35^{D620N/D620N}$ and WT mice at 6 or 10 months of age. Scale bar, 100 μm. Quantification of TH-positive neurons in SNpc (B,D) and OD of TH-positive fibers in striatum (C,E) of $VPS35^{D620N/D620N}$ and WT mice at 6 months old (B,C, n=6), 10 months old (D,E, n=6) . (F) Representative images of GFAP or IBA1 immunostaining in SNpc and striatum of $VPS35^{D620N/D620N}$ and WT mice at 6 or 10 months of age. Scale bar, 100 μm. (G-N) Quantification of GFAP positive cells (G-J) and IBA1 positive cells (K-N) in SNpc (G,I,K,M) and striatum (H,J,L,N) of 6-month-old (G,H,K,L) or 10-month-old (I,J,M,N) $VPS35^{D620N/D620N}$ and WT controls (n=6/genotype). data are shown as mean ± SEM; Student's t-test, unpaired, two tailed; n.s., not significant.

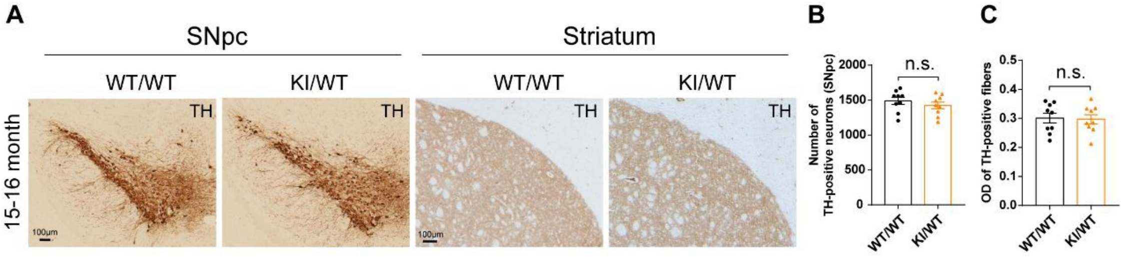

Figure S4. Lack of neuropathological changes in 15-16 months old heterozygous *VPS35* D620N KI mice. (A) Representative images of TH immunostaining in SNpc and striatum of *VPS35*$^{D620N/WT}$ and WT mice at 15-16 months old. Scale bar, 100 μm. (B) Quantification of TH- positive neurons in SNpc and (C) OD of TH-positive fibers in striatum of *VPS35*$^{D620N/WT}$ and WT mice at 15-16 months old (n=6). data are shown as mean ± SEM; Student's t-test, unpaired, two tailed; n.s., not significant.

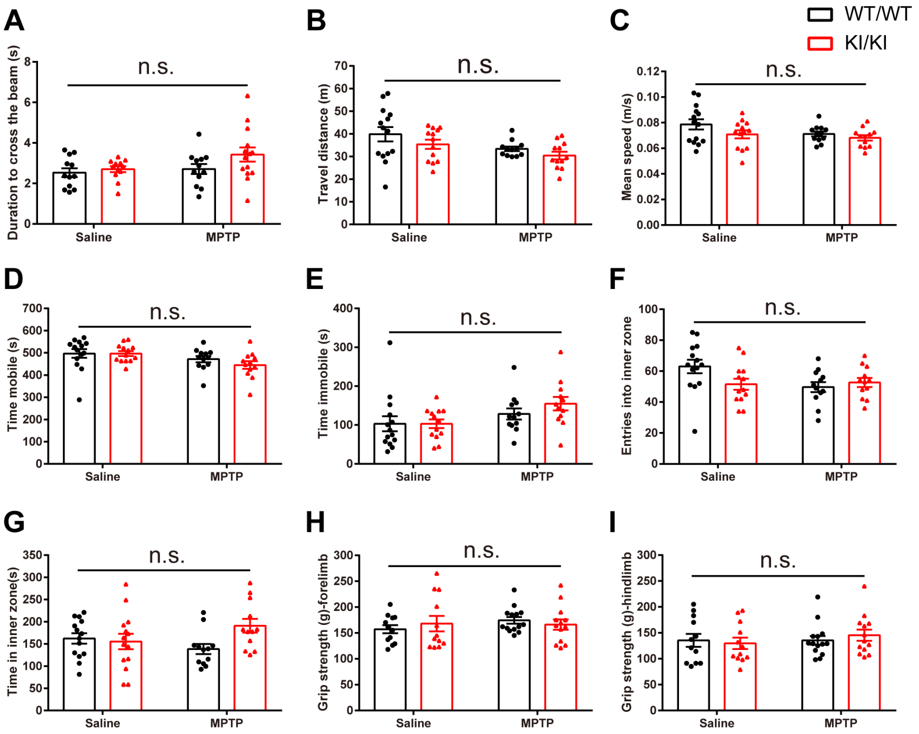

Figure S5. Behavioral analysis of $VPS35^{D620N/D620N}$ mice and littermate WT controls with MPTP/Saline treatment. (A) Duration to cross the wide beam (16mm) in beam walking test. (B-G) Travel distance, mean speed, Time mobile, time immobile, entries into inner zone and time in inner zone in open field test. (H) Forelimb grip strength. (I) Hindlimb grip strength. n=13-14/group; data are shown as mean ± SEM; n.s., not significant; two-way ANOVA with Tukey's post hoc test.

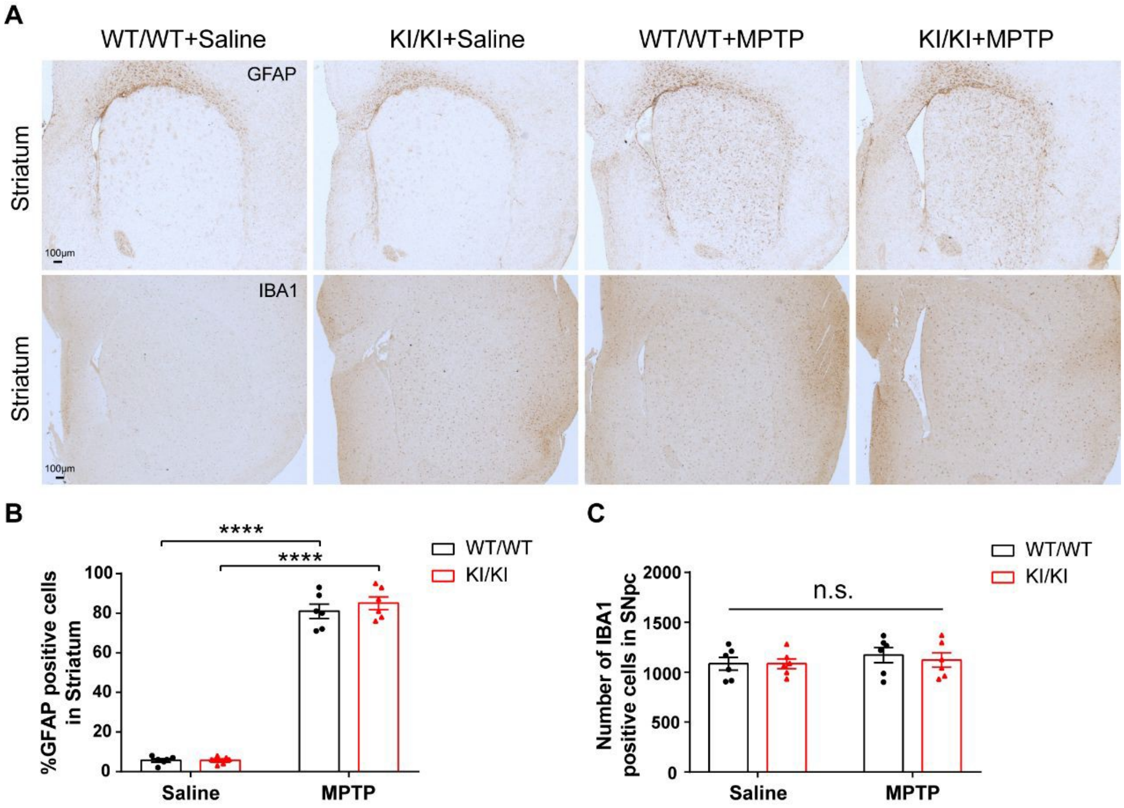

Figure S6. Analysis of neuroinflammation in striatum from *VPS35$^{D620N/D620N}$* mice and WT controls injected with MPTP/Saline. (A) Representative images of GFAP and IBA1 immunostaining in striatum from *VPS35$^{D620N/D620N}$* and WT mice injected with MPTP/Saline as indicated. Scale bar, 100 μm. (B) Quantification of %GFAP positive cells in striatum. (C) Quantification of IBA1 positive cells in striatum. n=6; mean ± SEM; two-way ANOVA with Tukey's post hoc test; *P < 0.05, **P < 001, ***P < 0.001, n.s., not significant.
