# Supplementary figures and images for: VPS35 D620N knockin mice recapitulate cardinal features of Parkinson’s disease
Source: Aging Cell. 2021 Mar 21;20(5):e13347. doi: 10.1111/acel.13347 (PMC8135078; doi:10.1111/acel.13347)

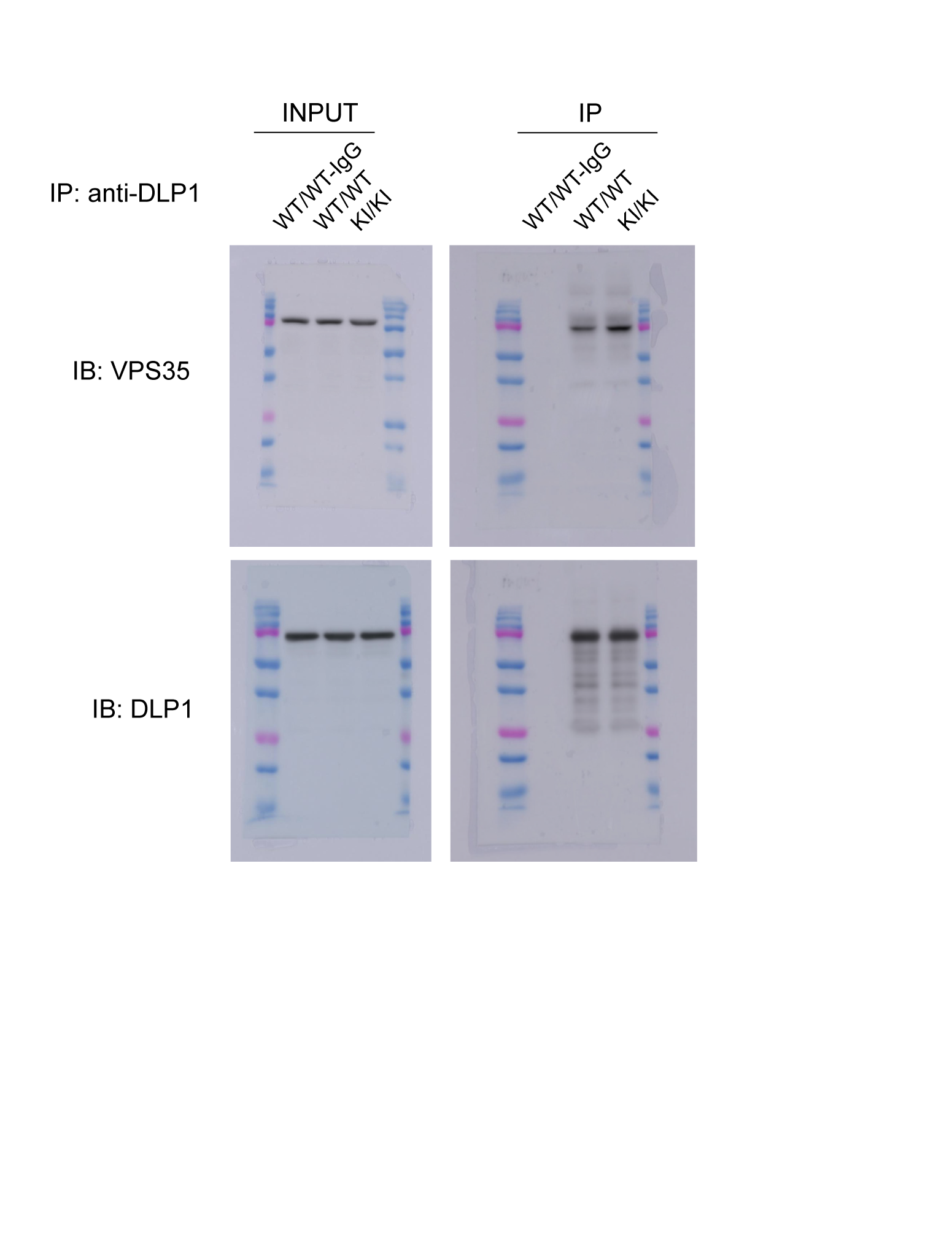

Supplement: Supplementary file 2 — Fig S7 [file ACEL-20-e13347-s002.tif]
